# Multi-Tissue Transcriptomic Profiling Identifies Anther Preferentially Expressed Candidate Genes in Cotton

**DOI:** 10.3390/biology15161433

**Published:** 2026-08-20

**Authors:** Juanjuan Feng, Hexuan Zhang, Xuexian Zhang, Huini Tang, Bingbing Zhang, Tingxiang Qi, Liping Guo, Chaozhu Xing, Jianyong Wu

**Affiliations:** State Key Laboratory of Cotton Bio-breeding and Integrated Utilization, Institute of Cotton Research of Chinese Academy of Agricultural Sciences, 38 Huanghe Dadao, Anyang 455000, China

**Keywords:** cotton, anther development, transcriptome, anther-preferentially expressed genes, gene expression profiling

## Abstract

Anther development is a complex and highly regulated process essential for pollen formation and reproductive development. However, systematic identification of genes exhibiting anther-preferential expression patterns through multi-tissue transcriptomic comparisons remains limited in cotton. In this study, comparative transcriptome analysis was performed using root, stem, leaf, and anther tissues of the cotton D8R line to identify candidate genes associated with anther development. Transcriptomic comparisons revealed extensive transcriptional differences between anthers and vegetative tissues, with 11,255 differentially expressed genes commonly identified among the three pairwise comparisons. Through stringent expression filtering, 843 putative anther-preferentially expressed candidate genes were identified, showing predominant expression in anthers and minimal expression in vegetative tissues. Nine representative genes, including *GhGDSL_A08_*, *GhGDSL_D08_* esterase/lipase, *GhPRX* (class III peroxidase), *GhAGL* (MADS-box transcription factor), and *GhTKPR1-like* reductase genes, were further validated by qRT-PCR, confirming their anther-preferential expression patterns. Promoter analysis of the nine validated genes identified three recurrent sequence motifs among seven selected promoters, including CG-rich and G-rich sequences, which showed similarity to reported binding motifs of *CAMTA*, *bHLH*, and *TCP* transcription factors. Collectively, this study provides a valuable transcriptomic resource of anther-preferentially expressed candidate genes and potential cis-regulatory features, offering genetic resources for further investigation of cotton anther development and related reproductive processes.

## Simple Summary

Anther development is a complex process controlled by the coordinated activity of many genes, but genes preferentially expressed in cotton anthers have not been systematically identified. In this study, gene expression profiles were compared among roots, stems, leaves, and anthers of cotton to identify candidate genes with preferential expression in anthers. A total of 843 candidate genes were identified, and the expression patterns of nine representative genes were further confirmed by quantitative analysis. We also examined DNA sequences near these nine genes and identified recurring sequence features that may provide clues to their expression regulation. This study provides a useful resource of candidate genes for further investigation of cotton anther development.

## 1. Introduction

Cotton is an economically important natural fiber crop that plays a pivotal role in the global textile industry [1]. Improving yield potential, fiber quality, and stress adaptability remains a major objective in cotton breeding programs. In addition to agronomic traits, reproductive development is a critical factor determining seed production and hybrid breeding efficiency. Normal anther development and pollen formation are essential for maintaining male fertility and ensuring successful reproduction in cotton. Previous studies have demonstrated that defects in anther development and pollen formation can lead to male sterility, which has been utilized as an important genetic resource for hybrid seed production in crops [2]. Therefore, systematic identification of genes preferentially expressed in anthers is essential for understanding the molecular basis of anther development and provides valuable candidate resources for future studies of reproductive regulation and cotton improvement.

Anther development is a highly coordinated physiological, biochemical, and morphogenetic process, requiring precise regulation of complex transcriptional networks involving numerous genes associated with carbohydrate metabolism, hormone homeostasis, lipid biosynthesis, and other metabolic pathways [3]. Aberrant expression of genes involved in microsporogenesis can severely disrupt normal microspore development and represents a major cause of male sterility in plants [4]. Although high-throughput RNA sequencing (RNA-seq) approaches have been widely applied to investigate transcriptomic differences between fertile and sterile anthers (or flower buds) in cotton CMS systems [5,6,7], most previous studies have focused primarily on comparisons within a single tissue, particularly anthers. The widespread expression of housekeeping genes and associated background transcriptional noise has remained a major challenge for accurately identifying anther-preferential predicted regulatory modules in the CMS-D8R system. In rice and wheat, comparative transcriptome analyses incorporating vegetative tissues as controls have successfully facilitated the identification of tissue-specific regulatory factors [8,9]. However, to date, comprehensive multi-tissue comparative transcriptomic studies aimed at systematically deciphering anther-preferential expression profiles in the cotton D8R system have not been reported.

The establishment of male fertility in flowering plants depends on precisely orchestrated transcriptional regulation and cell fate determination during anther and pollen development [10,11]. Accumulating evidence has revealed multiple gene families that exert essential functions during plant anther development. In *Arabidopsis*, the functionally redundant class III peroxidases *PRX9* and *PRX40* maintain tapetal cell wall integrity by cross-linking extensins, and their simultaneous loss results in complete male sterility [12]. In rice, *GDSL*-type lipases participate in lipid remodelling within anthers, and loss-of-function mutations of anther-specific *GDSL* genes disrupt tapetum differentiation and pollen exine formation, eventually leading to male sterility [13]. In maize, two tetrapeptide α-pyrone reductases, *ZmTKPR1-1* and *ZmTKPR1-2*, function as key enzymes in sporopollenin biosynthesis and are essential for pollen exine formation. Loss of both genes results in defective pollen wall development, delayed tapetum degeneration, and complete male sterility, highlighting their critical roles in pollen development and fertility [14]. It has been reported that suppression of *AGL6* expression in *Isatis indigotica* and wheat (*Triticum aestivum*) leads to reduced stamen number and aberrant anther development [15,16]. In cotton, several genes affecting anther development have been identified, including *GhGPAT12/25*, which regulate tapetum degradation, anther cuticle formation, and pollen exine development [17]; *GhTKPR1_8*, which functions in sporopollenin biosynthesis and affects anther dehiscence and pollen viability [18]; and *Gh4CL20/20A*, which ensures proper anther development by regulating flavonoid metabolism [19]. However, despite the functional characterization of several anther-specific genes in other crops, a systematic identification of such genes through multi-tissue comparative transcriptomics has not been performed in cotton.

To systematically identify genes associated with anther development, this study performed a comparative multi-tissue transcriptomic analysis of the cotton D8R line using roots, stems, leaves, and anthers. Through stringent expression filtering, 843 putative anther-preferentially expressed candidate genes were identified, among which nine representative genes were further validated by qRT-PCR. In addition, recurrent promoter motifs among selected candidate genes provided preliminary insights into the potential cis-regulatory features underlying anther-preferential expression. Collectively, this study establishes a valuable transcriptomic resource and provides candidate genes for future investigation of anther development and related reproductive processes in cotton.

## 2. Materials and Methods

### 2.1. Plant Material Collection

Cotton D8R is a stable, homozygous restorer line with normal anther development and male fertility, providing a well-defined genetic background for identifying genes preferentially expressed in fertile anthers while minimizing the influence of genetic heterogeneity. At the seedling stage, young and fresh true leaves and stems of cotton D8R were harvested, immediately placed into 5 mL RNase-free cryogenic tubes, and promptly frozen in liquid nitrogen. For root samples, roots were first rinsed with running tap water to remove adhering impurities, then washed with 75% ethanol to eliminate surface soil microorganisms, and finally blotted dry with absorbent paper to remove residual ethanol. The cleaned roots were then placed into 5 mL RNase-free cryogenic tubes and immediately snap-frozen in liquid nitrogen. For each tissue (root, stem, leaf, and anther), three independent biological replicates were collected for RNA extraction and transcriptome sequencing.

At the squaring stage, flower buds measuring 1–5 mm in length were collected from CMS-D8R plants. Anthers were carefully dissected from the buds on ice, immediately transferred into 2.0 mL RNase-free microcentrifuge tubes, flash-frozen in liquid nitrogen, and stored at −80 °C until RNA extraction. The selected buds encompass the major developmental window of cotton anther development and were used for both RNA-seq and qRT-PCR analyses.

### 2.2. RNA-Seq

#### 2.2.1. RNA-Seq Library Construction and Sequencing

Total RNA was extracted from the root, stem, and leaf tissues of cotton D8R, and samples with high RNA integrity were selected for RNA-Seq library construction. Poly(A) mRNA was purified from total RNA using Oligo (dT) magnetic beads, fragmented into ~200 bp fragments, and reverse-transcribed into double-stranded cDNA. Following PCR amplification and purification with AMPure XP Beads (Beckman Coulter Life Sciences, Indianapolis, IN, USA), paired-end sequencing libraries were constructed. The libraries were quantified with a Qubit 2.0 fluorometer (Thermo Fisher Scientific, Waltham, MA, USA), diluted to 1.5 ng/µL, and validated for insert size via an Agilent 2100 Bioanalyzer (Agilent Technologies, Inc., Santa Clara, CA, USA). Effective concentrations were further determined by qRT-PCR for quality control. All libraries were subsequently sequenced on the DNBSEQ-T7 platform (MGI Tech Co., Ltd., Shenzhen, China) using the paired-end 150 bp sequencing strategy. The transcriptome datasets analyzed in this study were all generated from the cotton D8R. The anther RNA-seq dataset was generated in our previous study and deposited in the NCBI BioProject database under accession number PRJNA685585. The root, stem, and leaf RNA-seq datasets were newly generated in the present study and have been deposited under the same BioProject accession (PRJNA685585). These datasets were analyzed using the same reference genome and bioinformatic pipeline for comparative transcriptome analysis among different tissues.

#### 2.2.2. RNA-Seq Data Quality Control

Raw sequencing reads contained adapter sequences, low-quality bases, and ambiguous N bases, which could interfere with subsequent bioinformatic analyses. To obtain high-quality clean reads, raw data from each sample were filtered using SOAPnuke (v2.1.0) [20] with the key parameters –lowQual = 20, –nRate = 0.005, and –qualRate = 0.5, while all other parameters were set to default values. The detailed filtering criteria were as follows: removal of paired reads carrying adapter sequences, elimination of paired reads with an N base ratio above 0.5%, and exclusion of low-quality paired reads where more than 50% of bases had a Qphred value ≤ 20.

High-quality clean reads were aligned to the D8R reference genome using Hisat2 (v2.1.0) [21]. To quantify transcript abundance, Bowtie2 (v2.3.5) [22] was used to map clean reads to a comprehensive transcript dataset, which integrated the annotated transcripts of the D8R reference genome. The RNA-seq data for all samples showed Q30 values exceeding 90%, and more than 95% of the clean reads were successfully mapped to the reference genome (Supplementary Table S1).

### 2.3. Gene Expression Quantification and Differential Expression Analysis

Gene expression levels were quantified using RSEM (v1.3.1) [23] based on Bowtie2 alignment results [22]. Raw read counts were obtained for each transcript, and FPKM (Fragments Per Kilobase of transcript per Million mapped reads) values were computed for the normalization of gene expression levels [24]. Paired-end reads derived from the same fragment were counted as a single fragment. FPKM values were used to visualize gene expression patterns and to screen anther-preferentially expressed genes. Principal component analysis (PCA) was performed using the prcomp function in R (version 4.2.2). Pearson correlation coefficients were calculated from the FPKM expression matrix using the cor function with the Pearson method, and hierarchical clustering was performed to assess sample similarity (Figure S1).

Differentially expressed genes (DEGs) were identified using DESeq2 (v1.22.2) [25]. Raw read counts were used as input, with the experimental design specified as design = ~ group, where group represents the tissue type. Separate pairwise comparisons were performed between anthers and each vegetative tissue (anther vs. root, anther vs. stem, and anther vs. leaf). *p* values were adjusted using the Benjamini–Hochberg method, and genes with adjusted *p* < 0.05 and |log2FC| > 1 were considered DEGs. FPKM values were used only to visualize gene expression patterns and were log_2_-transformed as log_2_(FPKM + 1) for heatmap visualization.

### 2.4. GO and KEGG Enrichment Analysis

To investigate the functional characteristics of the identified DEGs, Gene Ontology (GO) annotation and Kyoto Encyclopedia of Genes and Genomes (KEGG) pathway enrichment analyses were performed using the clusterProfiler package (v4.0.0) in R. For enrichment analysis, the background gene set consisted of all cotton genes with available GO annotations or KEGG pathway annotations, respectively. Over-representation analysis was performed using the hypergeometric test (phyper). The resulting P-values were adjusted for multiple testing using the Benjamini–Hochberg false discovery rate (FDR) method. GO terms and KEGG pathways with an FDR ≤ 0.05 were considered significantly enriched. The enriched GO terms and KEGG pathways were visualized using clusterProfiler and are presented as bar charts, with the number of DEGs assigned to each functional category or pathway shown.

### 2.5. Analysis of Expression Pattern Clustering and Identification Anther-Preferentially Expressed Genes

To identify genes with similar expression patterns across different tissues, expression pattern clustering analysis was performed using the TCseq package in R (version 4.2.2). The normalized expression matrix of the 11,255 DEGs was standardized (standardize = TRUE) before clustering. Genes were classified into nine clusters (k = 9) using the C-means clustering algorithm (algo = “cm”), and Euclidean distance (dist.method = “euclidean”) was used to calculate the similarity between expression profiles. The resulting clusters were used to characterize tissue-preferential expression patterns.

For each gene, mean FPKM values were calculated from three biological replicates within each tissue (root, stem, leaf, and anther). These mean values were then used to apply the three filtering criteria: (i) mean FPKM < 2 in all vegetative tissues; (ii) mean FPKM ≥ 2 in anthers; and (iii) the ratio of mean FPKM in anthers to that in each vegetative tissue was at least 10. This approach reduces the influence of inter-replicate variation and provides a stable estimate of gene expression levels for each tissue.

### 2.6. Quantitative Real-Time Reverse Transcription PCR Analysis

The expression patterns of selected candidate genes were validated by quantitative real-time PCR (qRT-PCR). Total RNA used for qRT-PCR was obtained from the same RNA preparations used for RNA-seq analysis. First-strand cDNA was synthesized using the PrimeScript™ RT Reagent Kit (TaKaRa, Beijing, China) according to the manufacturer’s instructions. qRT-PCR was performed using TransStart^®^ Top Green qPCR SuperMix (TransGen Biotech, Beijing, China) on a real-time PCR system following the manufacturer’s protocol. The thermal cycling conditions were as follows: 94 °C for 30 s, followed by 45 cycles of 94 °C for 5 s and 56 °C for 15 s. Following amplification, melting-curve analysis was performed to verify the specificity of each primer pair. All reactions produced a single distinct melting peak without evidence of non-specific amplification or primer-dimer formation. The cotton *His3* gene was utilized as an internal control [26]. The 2^−ΔΔCT^ method was used to calculate the relative gene expression levels [27]. Each analysis included three independent biological replicates, and each biological replicate was analyzed with three technical replicates. Primer sequences are listed in Supplementary Table S7.

### 2.7. Promoter Motif Analysis

The 2-kb upstream promoter sequences of the nine anther-preferentially expressed genes were extracted from the reference genome. Recurrent sequence motifs were identified using the MEME Suite (https://meme-suite.org/meme/tools/meme, accessed on 14 June 2026) in Classic mode with the Zero or One Occurrence Per Sequence (ZOOPS) model, while all other parameters were kept at their default settings. Sequence similarity between the identified motifs and previously reported transcription factor-binding consensus sequences was subsequently examined by manual comparison with published motif information to identify potential matches. These comparisons were used to provide preliminary predictions and did not constitute experimental evidence of transcription factor binding or motif enrichment.

## 3. Results

### 3.1. Screening of DEGs in Different Tissues of D8R

**Figure 1:**
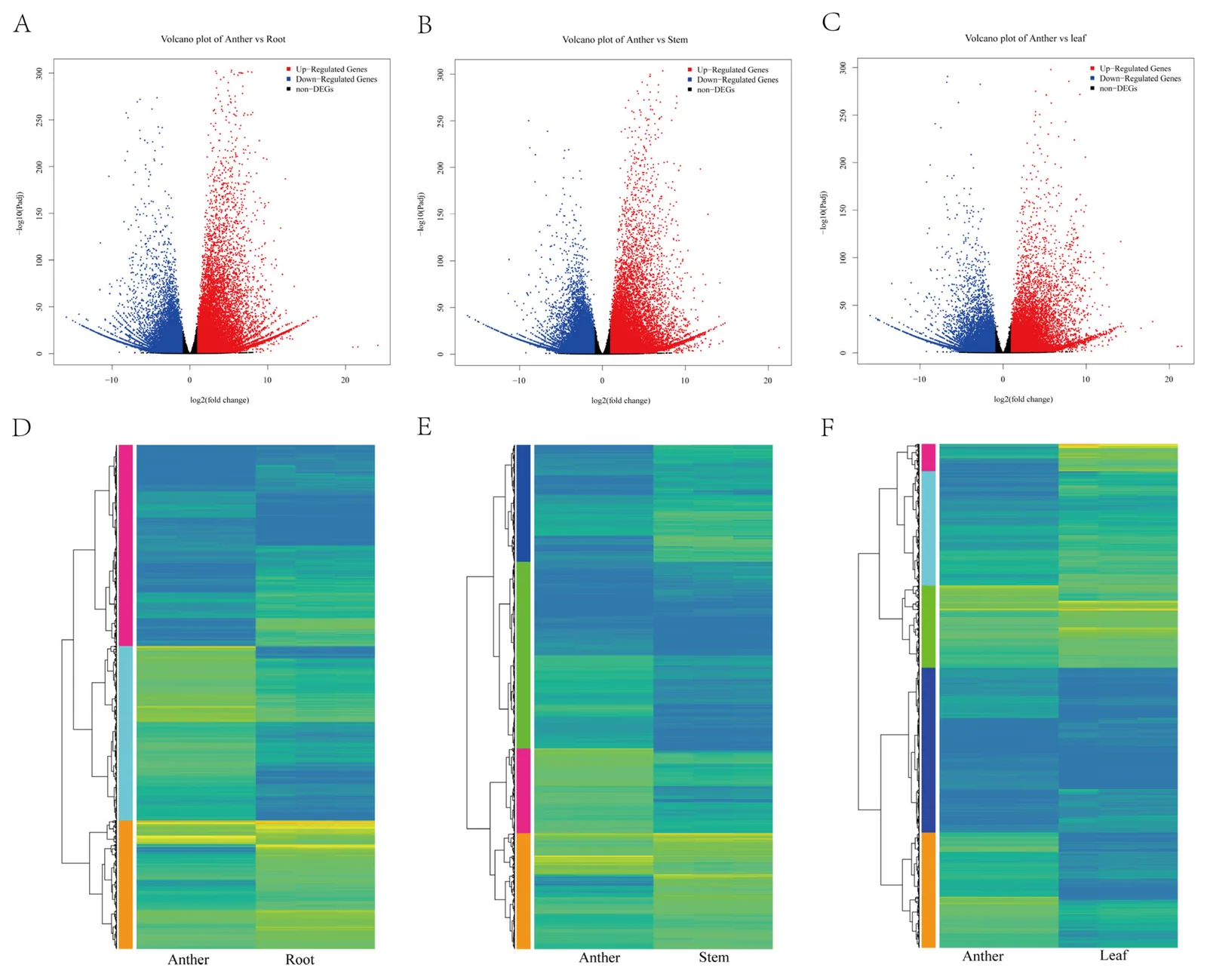
Transcriptomic landscape of DEGs in D8R tissues. (**A**–**C**) Volcano plots showing the distribution of DEGs in anther vs. root (**A**), anther vs. stem (**B**), and anther vs. leaf (**C**). Up- and down-regulated genes are highlighted in red and blue, respectively. (**D**–**F**) Heat maps visualizing the expression profiles of DEGs across anther and the three vegetative tissues—root (**D**), stem (**E**), and leaf (**F**)—with color scales representing relative expression levels.

To investigate the transcriptional differences between anthers and vegetative tissues of the D8R restorer line, we performed RNA-seq analysis on root, stem, leaf, and anther samples, generating three pairwise comparison groups: anther versus root (A-vs.-R), anther versus stem (A-vs.-S), and anther versus leaf (A-vs.-L). As presented in Figure 1A, the A-vs.-R comparison yielded the largest number of DEGs, with 29,431 identified, of which 15,217 were upregulated and 14,214 were downregulated in anther relative to root (Table S2). In the A-vs.-S comparison, a total of 29,122 DEGs were detected, comprising 15,207 upregulated and 13,915 downregulated genes (Figure 1B, Table S2). The A-vs.-L comparison revealed 22,361 DEGs, including 12,637 upregulated and 9724 downregulated genes (Figure 1C, Table S2). In total, 42,071 unique DEGs were identified across all three comparisons.

To visualize the expression dynamics of these DEGs across tissues, we generated heat maps using the R software (Figure 1D–F). As shown in Figure 1D–F, the clustering patterns clearly distinguished anther from the three vegetative tissues, with each tissue displaying a distinct transcriptional profile.

### 3.2. GO and KEGG Analysis of DEGs in Different Tissues from D8R

**Figure 2:**
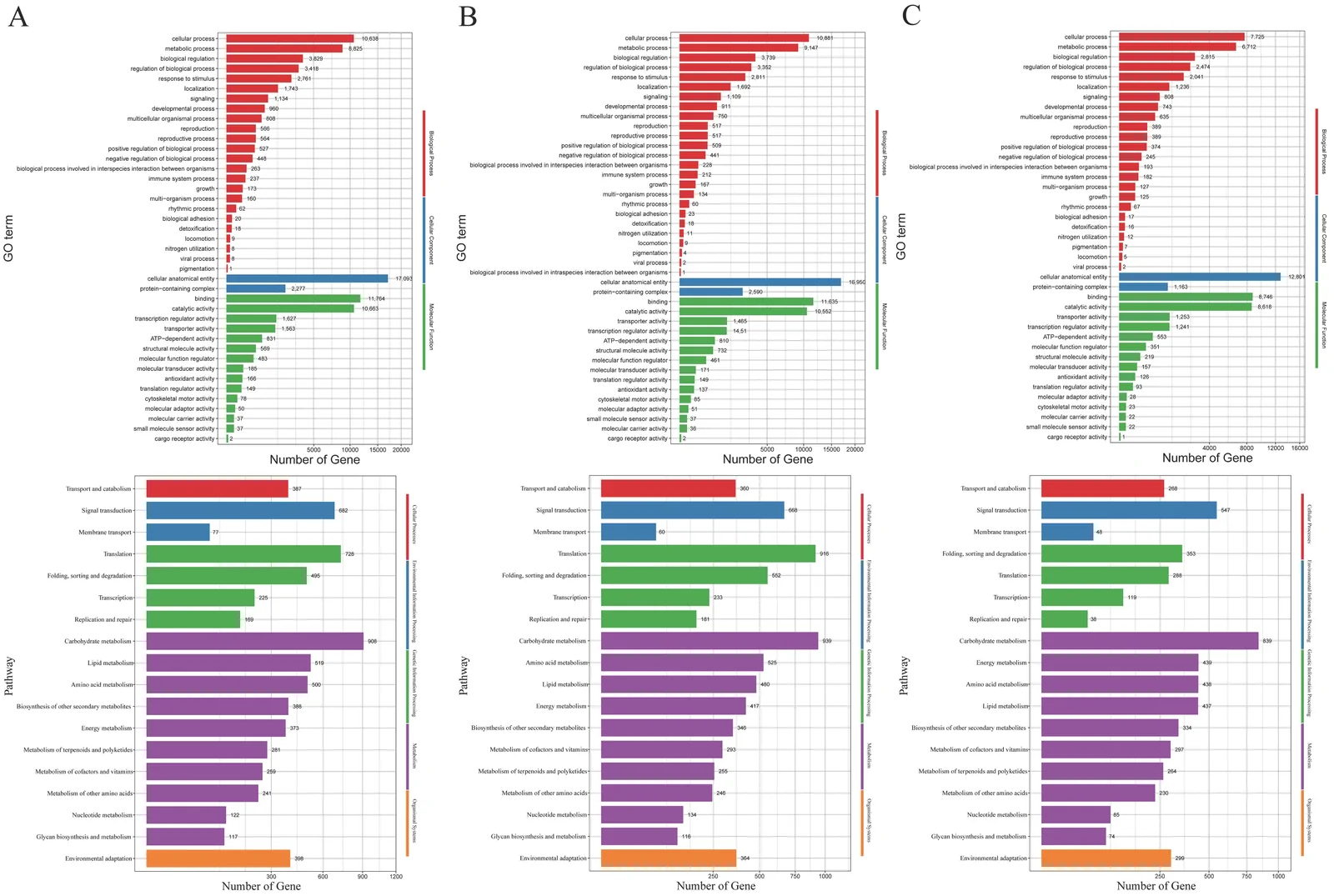
GO functional annotation and KEGG pathway enrichment analysis of DEGs from three comparative groups. (**A**–**C**) GO enrichment analysis of DEGs for anther vs. root (**A**), anther vs. stem (**B**), and anther vs. leaf (**C**). The histogram displays the enriched top GO terms covering biological process, cellular component, and molecular function categories, with bar lengths representing the numbers of enriched DEGs. (**D**–**F**) KEGG pathway enrichment analysis of DEGs for anther vs. root (**D**), anther vs. stem (**E**), and anther vs. leaf (**F**).

To explore the biological functions of the identified DEGs, GO annotation and KEGG pathway enrichment analyses were performed for three comparative groups (anther vs. root, anther vs. stem, and anther vs. leaf) (Figure 2). Although all groups exhibited similar functional classification patterns, the number of DEGs followed the order of anther vs. stem > anther vs. root > anther vs. leaf. In GO enrichment, cellular process and metabolic process predominated in the biological process category, while cellular anatomical entity and protein-containing complex were the most enriched terms in the cellular component category. Distinct molecular functional preferences were observed across groups: catalytic activity was most enriched in the anther vs. root comparison, whereas binding activity was predominant in both the anther vs. stem and anther vs. leaf groups. KEGG pathway analysis identified phenylpropanoid biosynthesis, plant hormone signal transduction, and starch and sucrose metabolism as commonly enriched pathways among the three comparisons. Although these pathways are commonly identified in comparative transcriptomic analyses of plant tissues, they are also closely associated with essential biological processes during anther development.

### 3.3. Analysis of Commonly Differentially Expressed Genes Between Anther and Other Tissues

**Figure 3:**
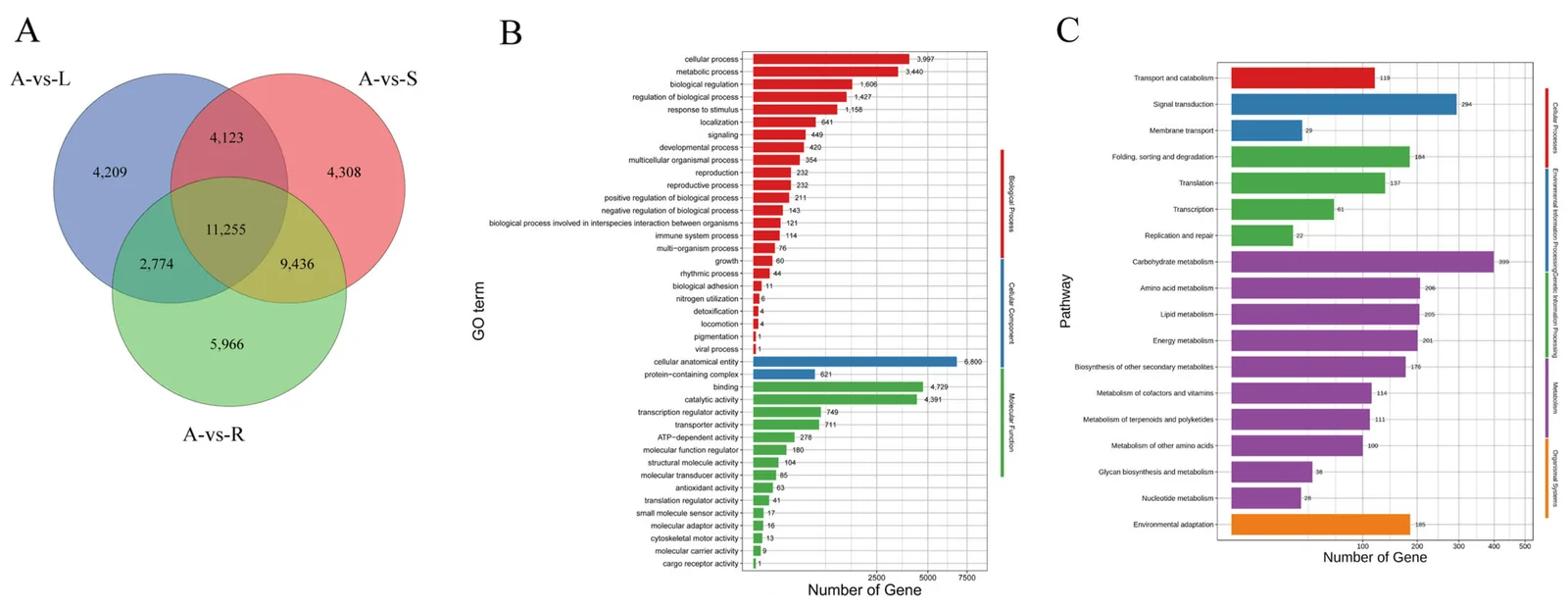
DEGs and functional enrichment across tissue comparisons in D8R. (**A**) Venn diagram of DEG overlaps among comparison groups. (**B**) GO annotation of DEGs. (**C**) KEGG pathway analysis of DEGs.

To characterize the transcriptomic landscape of the D8R restorer line and identify genes preferentially expressed in anthers, we performed pairwise differential expression analyses between anther (A) and each of the three vegetative tissues, namely root (R), stem (S), and leaf (L). A total of 11,255 genes were identified as differentially expressed in anther simultaneously in pairwise comparisons with all three vegetative tissues (Figure 3A). Gene Ontology (GO) functional annotation of these 11,255 DEGs revealed that, in the Biological Process category, 3997 genes were assigned to “cellular process” and 3440 genes to “metabolic process”. In the Cellular Component category, 6800 genes were annotated to “cellular anatomical entity”. In the Molecular Function category, 4729 DEGs were associated with “binding” (Figure 3B). KEGG pathway analysis further suggested that 201 DEGs were involved in “energy metabolism” and 399 DEGs in “carbohydrate metabolism” (Figure 3C). Among the 11,255 DEGs, a total of 4523 genes were upregulated in the anther relative to vegetative tissues. Collectively, these results provide a transcriptomic overview of gene expression differences between anthers and vegetative tissues in D8R.

### 3.4. Expression Pattern Clustering of Common DEGs Across D8R Tissues

**Figure 4:**
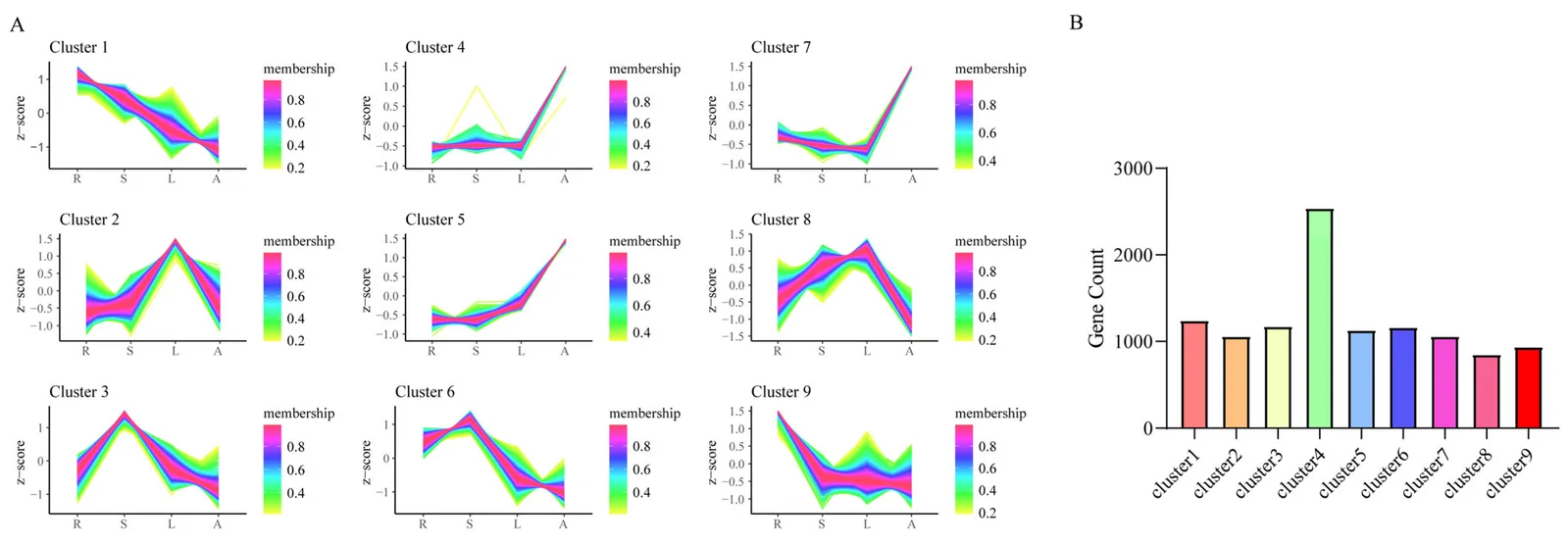
Expression pattern clustering of 11,255 DEGs across four tissues of cotton D8R. (**A**) Nine distinct expression clusters generated based on expression trend profiling. (**B**) Distribution of DEGs among the nine clusters. R: root; S: stem; L: leaf; A: anther.

A total of 11,255 DEGs identified among the four tissues of D8R were categorized into nine distinct expression pattern clusters (Figure 4A). The number of genes assigned to each cluster varied considerably (Figure 4B). Cluster 4 contained the largest gene set, comprising 2542 DEGs, whereas Cluster 8 represented the smallest cluster with 850 genes. Notably, Clusters 4 (2542 genes), 5 (1131 genes), and 7 (1061 genes) exhibited relatively high expression in anthers compared with vegetative tissues, with Cluster 4 showing the most pronounced anther-preferential expression pattern (Tables S3 and S4). Therefore, genes assigned to Cluster 4 were considered promising candidates for further analysis of anther development in cotton.

### 3.5. Identification of Anther-Preferentially Expressed Genes

**Figure 5:**
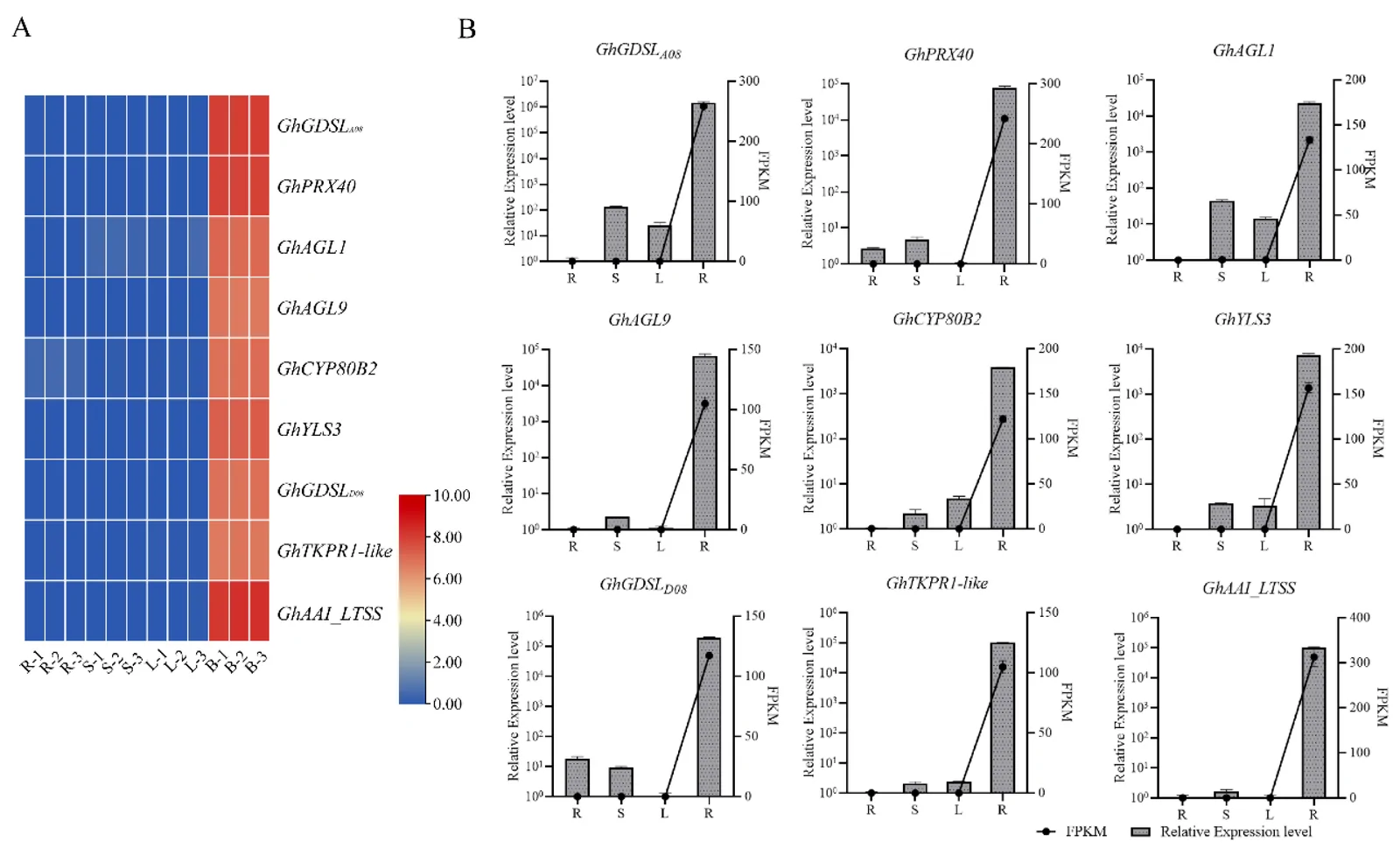
Expression profiling of nine anther-preferentially expressed candidate genes in different tissues of the CMS-D8 restorer line. (**A**) Heatmap showing transcript abundance of nine candidate genes based on multi-tissue transcriptome data. Expression values were normalized as log_2_(FPKM + 1) before visualization. R: root; S: stem; L: leaf; A: anther. Numbers 1–3 represent three biological replicates. (**B**) qRT-PCR validation of tissue expression patterns. Relative expression levels were determined in R, S, L, and A tissues. Error bars represent the standard error of three biological replicates.

Based on multi-tissue transcriptome data from the cotton D8R line, including roots, stems, leaves, and anthers, anther-preferentially expressed genes were identified by applying three simultaneous criteria: (i) FPKM < 2 in all vegetative tissues (roots, stems, and leaves); (ii) FPKM ≥ 2 in anthers; and (iii) transcript abundance in anthers was at least 10-fold higher than that in each vegetative tissue. A total of 843 candidate genes met these criteria (Tables S5 and S6), of which 806 (95.6%) were assigned to Cluster 4, which exhibited high expression in anthers and low or undetectable expression in vegetative tissues. The substantial overlap between the two datasets indicates a high degree of consistency between the expression filtering strategy and the clustering analysis. Among the 20 genes with the highest transcript abundance in anthers, 11 encoded hypothetical proteins without functional annotation, whereas the remaining nine were functionally annotated and selected for further expression analysis. The expression profiles of the nine candidate genes, based on RNA-seq FPKM values, are presented as a heatmap (Figure 5A). Consistent with the screening criteria, all nine genes exhibited negligible transcript abundance in vegetative tissues but markedly higher expression in anthers. To further validate the RNA-seq results, anther-preferential expression patterns were examined by qRT-PCR (Figure 5B). The qRT-PCR results were consistent with the RNA-seq data, confirming that all nine genes were predominantly expressed in anthers and showed only low or undetectable expression in vegetative tissues. Among the validated genes, *GhGDSL_A08_*, *GhGDSL_D08_*, and *GhTKPR1-like* exhibited the highest transcript accumulation in anthers. Overall, these results validate the reliability of the transcriptome analysis and confirm the anther-preferential expression of the selected candidate genes.

**Figure 6:**
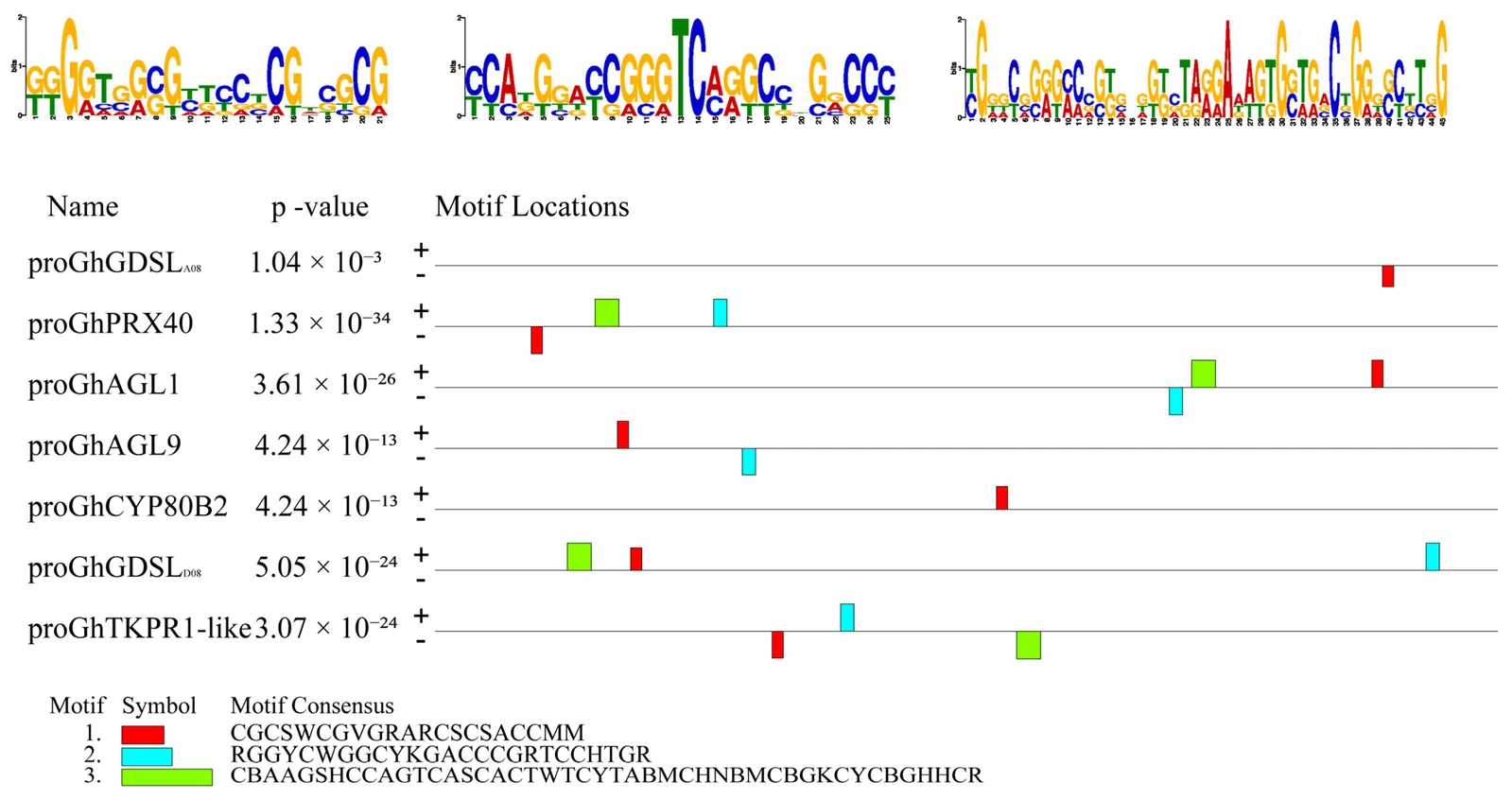
Recurrent sequence motifs identified among the selected promoters. Sequence logos of three recurrent sequence motifs are shown at the top. The middle panel illustrates the location of each motif on the promoter of seven selected candidate genes; “+” and “-“ represent positive and negative strands, respectively. At the bottom, the color symbols, motif ID and corresponding IUPAC consensus sequences of each motif are listed.

To investigate the potential cis-regulatory basis underlying anther-preferential gene expression, the 2-kb promoter sequences of the nine validated anther-preferentially expressed genes were analyzed using the MEME suite (https://meme-suite.org/meme/tools/meme, accessed on 14 June 2026). Three recurrent sequence motifs were identified among seven of the nine promoters (Figure 6). These motifs were distributed on both the positive (+) and negative (−) strands, with variable copy number among different promoters. Motif1 is characterized by tandem CG dinucleotides, whereas Motif2 is enriched in G-rich sequences. Motif3, the longest motif identified, contains substantial sequence degeneracy and shows no significant similarity to any previously reported cis-regulatory element. Sequence comparison suggested that Motif1 contains CG-rich fragments resembling the CAMTA-associated CG-box, whereas Motif2 contains G-rich regions with partial similarity to reported bHLH-binding and TCP-binding motifs. However, none of the three motifs contained complete canonical binding cores for these transcription factor families.

## 4. Discussion

Previous transcriptomic studies in cotton have mainly focused on developmental comparisons among anthers [28,29,30], which effectively identify stage-dependent genes but provide limited resolution for distinguishing constitutively expressed genes from high-confidence anther-preferential candidate genes. By incorporating vegetative tissues as references, the present study increased the stringency of candidate gene identification and provided a resource for further investigation of cotton anther development. Compared with previous single-organ transcriptome analyses, this cross-tissue comparative strategy reduced interference from ubiquitously expressed genes and enhanced the identification of genes associated with anther-preferential expression. The expression patterns of nine representative genes were further confirmed by qRT-PCR, validating the reliability of RNA-seq-based expression profiling. The use of pooled 1–5 mm flower buds may limit the resolution of stage-preferential expression patterns. Moreover, as the anther and vegetative datasets were generated separately, potential batch effects cannot be completely excluded.

Phenylpropanoid metabolism provides precursors for sporopollenin and other pollen wall components [31,32]; plant hormone signaling, including BR, GA, and JA pathways, regulates anther morphogenesis, tapetal function, and pollen development [33]; and starch and sucrose metabolism supplies the carbohydrates and energy required for pollen maturation [34]. Therefore, the consistent enrichment of these pathways reflects the active metabolic and developmental processes occurring in fertile anthers rather than serving as anther-preferential molecular signatures. To further characterize the potential functions of the identified anther-preferentially expressed candidates, nine representative genes with reliable functional annotations were selected for qRT-PCR validation. Functional annotation and homology analysis indicated that these candidates are potentially associated with biological processes related to anther development and pollen formation. GDSL lipases have been implicated in lipid metabolism associated with pollen exine formation, and their homologs are required for tapetum development and pollen fertility in rice [35]. Class III peroxidases participate in redox regulation and cell wall modification during anther development. In *Arabidopsis*, PRX9 and PRX40 contribute to microspore cell wall integrity [12], and altered peroxidase activity has been linked to abnormal tapetal development in cotton [36]. MADS-box transcription factors are known regulators of floral organ identity and anther development [37], while TKPR1 functions in sporopollenin biosynthesis, and disruption of its homologs affects pollen wall formation in rice and maize [14,38]. The functions of these cotton candidates require further experimental validation; their anther-preferential expression patterns and functional annotations provide valuable resources for future investigation of cotton anther development.

Cis-regulatory sequences within gene promoters may contribute to the regulation of gene expression. Promoter analysis identified three recurrent sequence motifs among seven of the selected promoters from the representative anther-preferentially expressed genes. Sequence comparison suggested that Motif1 contains CG-rich sequences reminiscent of the CAMTA-binding CG-box, whereas Motif2 contains G-rich sequences with partial similarity to reported bHLH-binding and TCP-binding motifs. These transcription factor families have been implicated in reproductive development and tapetum differentiation in several plant species [39,40,41]. However, because these assignments are based solely on sequence similarity and only a limited number of promoters were analyzed, the regulatory relationships remain speculative. Motif3 showed no obvious similarity to known transcription factor-binding motifs and therefore could not be functionally annotated in the present study. Collectively, these recurrent sequence motifs provide preliminary sequence-level clues for the regulation of the selected candidate genes, although their biological functions and interactions with upstream transcription factors require further experimental validation.

Overall, this study provides a comprehensive resource of anther-preferentially expressed genes and recurrent promoter sequence motifs in cotton, offering candidate genes for future studies of anther development and male fertility as well as potential genetic resources for molecular breeding. Further studies are required to validate the regulatory relationships between the predicted transcription factors and promoter motifs and to elucidate the biological functions of these candidate genes.

## 5. Conclusions

In this study, comparative multi-tissue transcriptome analysis of upland cotton identified 843 putative anther-preferentially expressed candidate genes through stringent expression filtering across root, stem, leaf, and anther tissues. The predominant anther expression patterns of nine representative candidates were further confirmed by qRT-PCR, supporting the reliability of the transcriptomic screening strategy. Functional annotation and expression validation suggested that these candidates may be involved in processes related to pollen development. In addition, recurrent sequence motifs were identified among selected promoters, providing candidate sequence features for further investigation of anther-preferential gene expression. Collectively, this study establishes a transcriptomic resource and provides candidate genes for further functional investigation of anther development.

## Data Availability

The RNA-seq data are available at NCBI SRA under accession PRJNA685585 (https://www.ncbi.nlm.nih.gov/bioproject/PRJNA685585, accessed on 16 June 2026).

## Supplementary Materials

The following supporting information can be downloaded at: https://www.mdpi.com/article/10.3390/biology15161433/s1. Figure S1: Principal component analysis (PCA) of transcriptome data from four tissues of cotton D8R; Table S1: RNA-seq quality control and alignment statistics for four tissues in cotton D8R; Table S2: Statistics of DEGs from anther-vegetative tissue pairwise comparisons; Table S3: List of gene IDs in clusters; Table S4: Gene counts of the nine expression clusters; Table S5: FPKM values of anther-preferentially expressed genes; Table S6: FPKM values of the 843 anther-preferentially expressed genes across three cotton genotypes; Table S7: The primers used in this study.
