# Supplementary material for: Multi-Tissue Transcriptomic Profiling Identifies Anther Preferentially Expressed Candidate Genes in Cotton
Source: Biology (Basel). 2026 Aug 20;15(16):1433. doi: 10.3390/biology15161433 (PMC13509745; doi:10.3390/biology15161433)
Supplement: Supplementary file 1 [file biology-15-01433-s001.zip › supplementary figure.pdf]

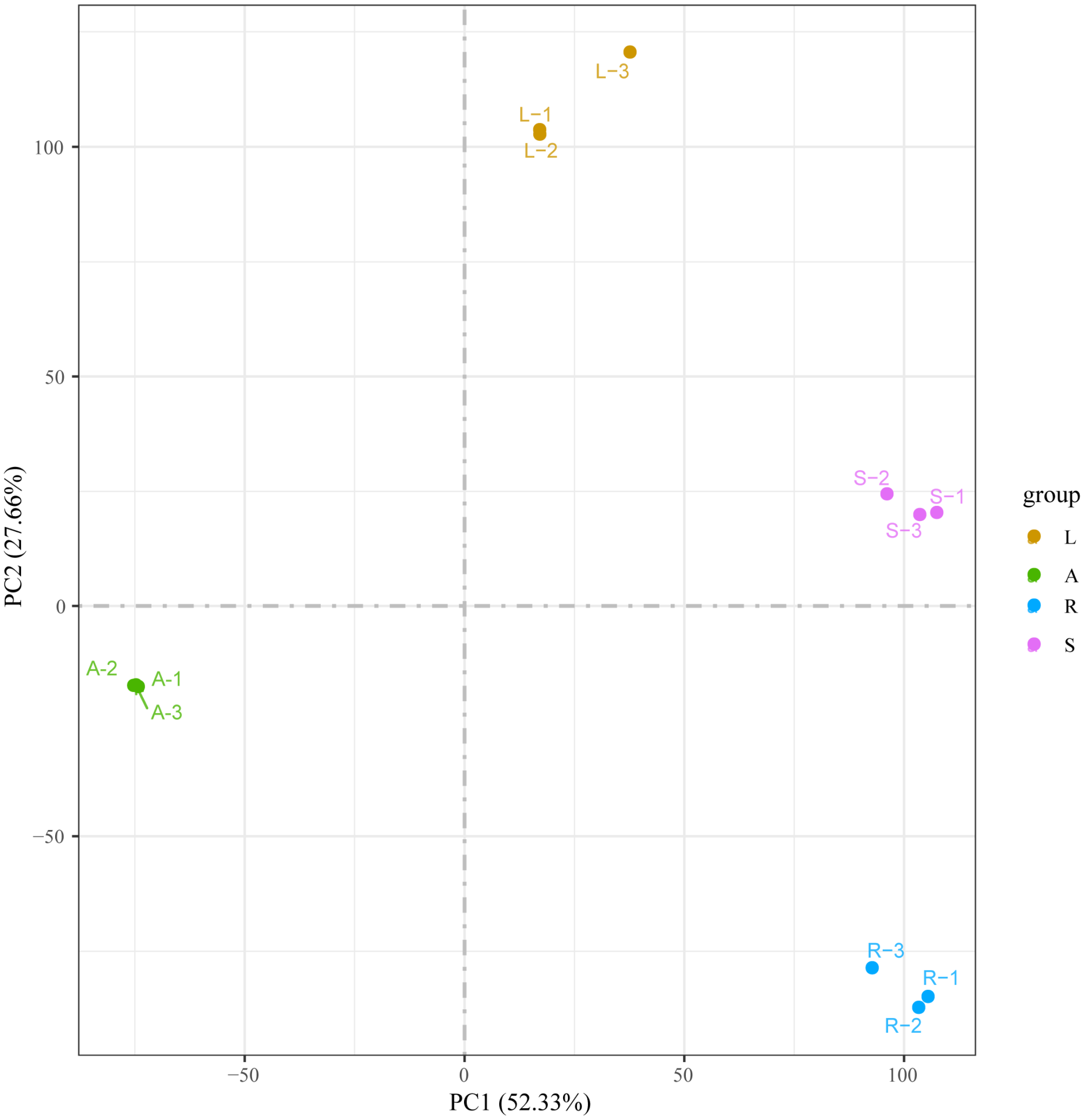

Figure S1. Principal component analysis (PCA) of transcriptome data from four tissues of cotton D8R.
